# How a Communication Intervention in Zambia Re-Oriented Health Services to the Needs of the Least-Supported

**DOI:** 10.3390/healthcare6030114

**Published:** 2018-09-13

**Authors:** Tony Klouda, Cathy Green, Miniratu Soyoola, Paula Quigley, Tendayi Kureya, Caroline Barber, Kenneth Mubuyaeta

**Affiliations:** 1 Freelance Consultant, 71A Lady Margaret Road, London NW5 2NN, UK; 2 Freelance Consultant, 28/4 Royal William Yard, Plymouth, Devon PL1 3GD, UK; cathygreenhpi@gmail.com; 3 DAI Global Health, Waterside Centre, North Street, Lewes, East Sussex BN7 2PE, UK; Miniratu_Soyoola@dai.com (M.S.); Paula_Quigley@dai.com (P.Q.); 4 Development Data, Plot 4 B, Lunzua Road, Off Addis Ababa, Rhodespark, Lusaka, Zambia; tendayi@developmentdata.info; 5 Transaid, 137 Euston Rd, Kings Cross, London NW1 2AA, UK; barberc@transaid.org; 6 Disacare, Plot # 11305, Off Chilimbulu Road, behind Libala High School, P.O. Box 50091 Lusaka, Zambia; kennymubuyaeta@gmail.com

**Keywords:** health communication, reaching every woman, social inclusion, universal health coverage, Zambia, maternal and newborn health, training, health workers

## Abstract

Despite decades of training health workers in communication, complaints from clients and communities about poor health worker attitudes abound. This was found to be so in Zambia where the More Mobilizing Access to Maternal Health Services in Zambia (MORE MAMaZ) program was trying to ensure the inclusion of under-supported women in a community-based maternal and newborn health program in five intervention districts. Under-supported women suffer a disproportionate burden of child mortality and are poor users of health services. An exploratory small-scale qualitative survey involving nurses from training schools and health facilities found that nurses knew how to communicate well, but were selective with whom and in what circumstances they did this. In general, those who received the worst communication were under-supported and had low confidence—the very people who needed the best communication. An experiential training program was started to help health workers reflect on the reasons for their poor communication. The training was evaluated after 14 months using semi-structured interviews and focus group discussions with staff at participating health facilities. The results showed improved inclusion of under-supported women but also increased attendance generally for ante-natal clinics, deliveries and under-five clinics. Another outcome was improved communication between, and a sense of job satisfaction among, the health workers themselves. The program demonstrated an effective way to improve the inclusion and involvement of the least-supported women and girls. There are important lessons for other health programs that aim to operationalize the goals of the Global Strategy for Women’s, Children’s and Adolescent’s Health, which include an emphasis on reaching every woman.

## 1. Introduction

Concerns about poor health worker communication are reflected in the recent call for respectful maternity care as part of the bid to improve maternal health [1,2]. Another important policy orientation is the current global commitment to reaching every woman, child and adolescent, ensuring that scarce health resources are targeted to where they are needed the most [3]. Against this backdrop, it is important to consider how health worker communication and the availability of respectful care facilitate or undermine efforts to reach the least-supported women and girls. 

It is widely understood that women who feel they are not respected or supported by their families or communities are less likely to care for themselves or their children—partly as a result of the lack of support, but also because of associated depression, anxiety and frustration [4,5,6,7,8]. They are also less likely to communicate well, and to remain silent or unquestioning when confronted with people who they perceive to be more powerful. This has important implications for health care access and a patient’s capacity to effectively communicate their needs or act on treatment and other advice when seeking health care. A recent study in northern Nigeria was pivotal in exposing the relationship between the burden of mortality and women’s lack of social support and voice, with important implications for health programs that are concerned with targeting resources to where they are needed the most [9].

In 2014, the Government of Zambia requested that an intervention to improve communication for nurses and other front-line health workers be included in a maternal and newborn health program called More Mobilizing Access to Maternal Health Services in Zambia (MORE MAMaZ). Operational from 2014 to 2016, the program was primarily a demand-side intervention focused on improving maternal and newborn health care access. Integrating the learning from the earlier study in Nigeria, the program placed significant emphasis on reaching the least-supported women in intervention communities. The request from the Ministry of Health (MOH) to design and implement a communications training intervention targeted to health workers was appropriate in a context where negative health worker attitudes were known to undermine community confidence in health services [10].

The communication training was implemented as a pilot and within an operations research framework with the intention of generating evidence to inform policy. The overall research objective was to assess the impact of the training on health workers’ awareness of how a lack of individual social support can be a key factor driving the skewed burden of ill-health. It also focused on health workers’ attitudes towards and capacity and willingness to support under-supported women in their catchment communities. Other objectives were to assess the impact of the training on the relationships between health workers within a health facility and on the linkages between health facilities and communities. This paper describes the development and implementation of the communications training and assesses its effectiveness in changing health workers’ understanding of the need to provide respectful care to all patients, whatever their background or situation.

## 2. Methods

In Zambia, the immediate impetus to train nurses and other front-line health workers in communication skills in the MORE MAMaZ intervention sites arose from persistent complaints from community members and health managers concerning the poor attitudes of health workers to clinic attenders. The concern was that this gap in the quality of care would impact negatively on the demand creation efforts that were underway at community level. Before beginning the training intervention, an effectiveness review of current communication training programs and a perspectives survey with health care workers were conducted. It was found that the Zambian nurse training program emphasized communication theory and practical applications similar to other programs around the world without taking into account the possibility that communication can be selective [11,12,13]. The underpinning assumptions were that not all health workers communicated badly; and those that did, communicated badly with all of their clients. However, there were no long-term evaluations of program effectiveness. Conversely, the pilot survey with Zambian healthcare workers found that while they knew how to communicate well, they applied these skills selectively. These findings challenged some long-standing assumptions about health worker communication skills.

### 2.1. Development of Communication Training

The understanding gained from the survey of nurse communication practices was used to plan a three-day training of health staff working in 64 health facilities in the program’s five intervention districts. The districts were Chama district in Muchinga Province; Mkushi, Serenje and Chitambo districts in Central Province; and Mongu district in Western Province. Curricula for core trainers (District Health Management Team (DHMT) members and nurse tutors) and for step-down training were devised with nurse tutors and Zambian consultants. The step-down training was designed to be delivered in two stages: first, a training of selected front-line health workers in participating health facilities, and, second, an orientation by the latter of other health workers in the facility, and also of Community Health Volunteers (CHVs) and other community members. The second group received a one-day step-down training. The training of CHVs and community members was important not only to help with the local identification of the least-supported women, but also to encourage those women to attend health services and to join groups. It also helped to better integrate the social inclusion work of the CHVs with that of the clinic-based health workers.

The program used an experiential training approach in which health workers reviewed themselves and developed insights into why they chose to communicate badly in some instances, whilst communicating well in others. The idea was that they would then be able to review how to manage and control the factors that led to instances of poor communication in their work setting. A starting point for reflection and discussion was the notion that the very people with whom health workers were most likely to communicate badly were those most likely to suffer ill health, or to have children who suffered ill health. The results of the nurse survey indicated that the use of general categories for targeting (e.g., ‘poverty’ or ‘lack of education’) was less useful than having an understanding of a client’s social situation at home. In other words, health workers needed to understand more about the underlying factors that led to a client presenting as poorly dressed, unwilling to communicate or shy. Health workers were also encouraged to think about the impact of poor communication on patients and on their colleagues—a topic not generally covered in formal health worker communication training. The need to improve communication between staff had not featured among the concerns cited by district health managers. This appeared to be a neglected area in supervision and management systems generally. The essential steps in the three-day training were:

- Reviewing the links between clinic use, mortality, deliveries and the support given to women.
- Exploring how social situations affect health.
- Analyzing why communication goes wrong with some people.
- Reflecting on how to guard against the temptation to be rude or dismissive.
- How to manage poor communication in the health facility as a team.
- Strategies for working with communities to promote the involvement of the least-supported women and girls through inclusive policies and participation in groups.

### 2.2. Training Evaluation

An evaluation of the training was undertaken in November 2015, 14 months after the first training input. The evaluation included 61 percent of participating health facilities (20 of 33 health facilities) in three of the five program districts: Chitambo, Serenje and Mkushi, all in Central Province. The evaluation was conducted with the full participation and involvement of the respective DHMTs.

Due to the staggered nature of the step-down training, health center staff, CHVs and community members received their training between seven and 12 months prior to the evaluation. A total of 20 health centers participated in the evaluation (eight in Chitambo, four in Serenje and eight in Mkushi). Semi-structured interviews were conducted with staff at the health centers, members of the DHMTs and the trainers. Focus group discussions were also carried out with CHVs and community representatives who were members of Neighborhood Health Committees. In each district, two teams of three interviewers conducted the interviews. A member of the DHMT was included on the interview team to improve relevance. In the health centers, 35 staff who had been trained (or oriented by other staff) were interviewed in addition to 22 CHVs. For 19 of the 20 health centers, a selection of community members (10–12 in each group) was also interviewed.

The interview questionnaires were designed separately for core trainers, health facility staff, CHVs, and community members. Each questionnaire had two components. The first set of questions was asked directly of interview respondents. These were fully open-ended to prevent bias towards a positive response. The answers were analyzed and coded by the evaluator for the main categories of response in relation to changes in communication, focus on under-supported women and changes in community support of the under-supported. The second set of questions was answered by the interviewer directly after the interview. These allowed the interviewer to check whether specific aspects of the training or experience had been mentioned, were implicit in the answers given by a respondent, or not mentioned. The answers were then appropriately coded. For this category of questions, the interviewer’s answers were checked against the respondent’s answers at the data analysis stage.

The teams of interviewers were given a one-day orientation on how to ask open-ended questions without giving any intimation of an expected answer. They also learnt how to assess what was implied in an answer, how to probe for fuller answers without directing them, and how to use the questionnaires. At the end of every day of interviewing, each team met to review the answers, and to provide their own feedback on what they had seen and heard.

Data was entered into Excel spreadsheets. Coded data was analyzed using the SPSS statistical package. A wide selection of the interviewee responses was provided in the evaluation. In order to contextualize the findings, the training evaluation examined in detail the government health statistics for each of the clinics visited, and compared these with areas where no training had taken place. For each area, the answers from the different types of respondents were cross-checked for consistency (e.g., the responses of clinic staff were checked against the perceptions of community members and CHVs).

The DHMTs from all intervention districts were invited to perform their own assessments of the training intervention before the evaluation. They were also invited to present these, together with their recommendations for future work, in a dissemination workshop involving donors, government representatives and other organizations.

Ethical approval was obtained as part of the overall approval given by the Zambian Ministry of Health for the operations research study as a whole.

There were few methodological limitations to the measurement of the impact of communications training to staff in clinics as the correlation between the answers of different staff (interviewed separately) at any particular clinic was very high. The clinics had been given no previous warning of the evaluation team visits, nor of the types of questions they would be asked. However, a failure to separate CHVs from other community representatives during focus group discussions meant that it was difficult to avoid positive bias in the answers as there was a tendency for the CHVs to answer in advance of others. This was dealt with by asking community members to answer first. However, this was not always successful.

## 3. Results

Thirteen core trainers were trained in September 2014. The core trainers trained 71 front-line health workers during September and October 2014. Over the next six months, front-line health workers oriented other health staff in their facility. The training was then extended to CHVs and other community representatives in the catchment area of participating health facilities. All training inputs were completed by April 2015.

The evaluation found that the communication training intervention was perceived to be successful in all but one health facility (a hospital outpatient clinic where staff turnover was very high) in relation to the improvement of communication in terms of: staff–staff relationships in health centers; communication between staff and their clients in general; a stronger, more nuanced and supportive focus on under-supported women in clinics; and improved linkages between health center staff, volunteers, community workers and communities. These improvements were described at every level (by DHMTs, health center staff, CHVs, community leaders and other representatives).

Almost all trainees who had received the three-day version of the training described how transformative the process had been for them as individuals. In general, they reported that it changed the way in which they understood people and approached their work. The majority of respondents amongst the clinic staff mentioned specifically that the training impacted them personally and significantly in terms of their understanding of why they communicated badly only with some people, and the realization that it was important to hide their frustrations from clients. In terms of improved communication within facility teams, one health worker explained:

“*The training has helped very much. It gave us an opportunity to think together as a team. Now people are happy to go to any staff rather than preferring to see only one. The way people interact, if we’re not on good terms, the client suffers. So we talk together.*”

Health workers reported that reviewing their experiences with other staff made communication easier.

Sensitivity to the social factors that affected women’s and girls’ health seeking behavior and responsiveness to treatment advice was also reported as having increased. As one health worker argued:

“*Before the training we did not bother to pay attention to the reasons for women’s circumstances, like looking dirty, unkempt and late for clinic. After the training we became more aware—we began to pay attention to these people to identify their situation better.*”

Overall, the general feedback from health workers was that the three-day communications training made their work more satisfactory and valued.

The communication training had a complementary and strengthening effect on the program overall. The work carried out by the program’s District Program Officers, who were embedded within the District Health Offices together with the District Mother and Child Health Coordinators, was seen to be invaluable in this regard. In many health facilities (with the backing of the DHMTs), health workers reported increased access to services by single pregnant women over and above that already achieved by the program. Although at first many clinic staff were wary of accepting pregnant women who had no partner because of the strong local antipathy to pregnancy outside of marriage, the awareness given by the training helped considerably in their acceptance of such women. In addition, clinic staff reported that, as a result of the training, they made more effort to improve the extent to which men accompanied such women to the health center. This resulted from their probing of women’s social situations and the fact that they then took time to find and interview partners (in instances where they had obtained the client’s consent).

Almost all the health facilities involved in the evaluation reported that attendance increased as a result of the training. It was reported that within the general increase in clinic attendance for institutional delivery, antenatal care and family planning services attendance by under-supported women also increased, as well as their attendance at general out-patients and under-five clinics.

The evaluation also identified a general increase in awareness of, and sensitivity to, the needs of under-supported women in the program intervention communities. Specific examples of improved support for women were given by 58 percent of community respondents. CHVs reported that their increased understanding of problems in their communication had further improved their ability to work with, assess and support the families of under-supported women.

Staff at the majority of the clinics reported that their training encouraged them to increase their probing of clients when they appeared to lack confidence, or when a client communicated badly. They reported that this encouraged many more women to talk frankly about their situations at home. They felt, in turn, that they were able to provide better advice to the women, and felt that this translated into a greater willingness to attend clinics, and better relations with the communities on social matters. It was reported in several of the health facilities that increased probing also helped women to talk about intimate partner violence. Although health staff were supposed to refer these cases to the police, after the training they reported that they spent more time counseling women, trying to interview domestic partners, and suggesting other forms of community support for the women.

## 4. Discussion

Much of the training given to health workers to date has been based on the assumption that health workers do not necessarily know how to communicate well, and that explaining the principles of communication will make them better communicators. The training intervention developed by MORE MAMaZ in Zambia was an attempt to approach the improvement of communication by other means—notably through internalization of the fact that people already know how to communicate well but choose when, and with whom, they communicate badly. The major success of the training was improved control of communication by health workers. This resulted not only from greater awareness of their choices, but also from the understanding that they could receive support from colleagues when trying to improve communication. The particular route chosen for this orientation was through demonstration of the impact of poor communication on those most likely to need good communication—notably the least supported women and girls in communities, those less likely to frequent health services as a result of that poor communication, and those most likely to neglect themselves and their children, and to suffer the highest mortality of their children.

Many of the headline results of the training evaluation were confirmed by the program’s statistical endline household survey, which was carried out in five intervention districts in 2016. The endline survey found that 73 percent of women and 70 percent of men in intervention communities were aware of efforts to include socially excluded women and girls in maternal and newborn health group activities. This was considerably more than in control sites (51 percent women and 61 percent of men) [14]. The endline survey also confirmed that intimate partner violence was perceived to have decreased across the program sites by 89 percent of male and 88 percent of female respondents [14]. The increase in facility attendance reported in the training evaluation was also confirmed. The endline survey identified a 25 percent increase in institutional delivery (from 64 percent to 89 percent), a 25 percent increase in antenatal care (ANC) attendance in the first trimester (from 37 percent to 62 percent), and a 14 percent increase in use of modern family planning methods (from 24 percent to 38 percent) [14]. The increases in service uptake were expected in relation to ANC and institutional deliveries, which had been major focal issues for the MORE MAMaZ program. Verbal reports by clinic-based staff and CHVs made clear their strong belief that more under-supported women were using health services as a result of the training. This appeared to be consistent with the evidence from the endline survey that first-time clinic attendance had increased. However, the lack of a reliable baseline that identified under-supported women separately from other women meant that there was no completely objective system of measurement of the increased attendance by under-supported women. It was also difficult to determine whether the work of CHVs, who had been trained by the program to seek out socially excluded women and girls and include them in maternal and newborn health group activities, or the work of health workers was more or less important in increasing facility attendance by individuals perceived locally to be under-supported. What can be said, however, is that the training intervention complemented other program activities to improve access by the least-supported women and girls. It is also likely that the focus on under-supported women within the program more generally, in combination with the communication training, increased the proportion of under-supported women using health services. Such difficulties in attributing change to a single intervention that is part of a broad-ranging, multi-focal program are not unusual, though they do point to the need to put in place robust monitoring and evaluation systems for pilot initiatives such as these if the aim is to prove their significance as an ‘innovation’.

The three-day training provided to clinic staff had considerable impact. By contrast, the one-day step-down training provided by clinic staff to colleagues and to CHVs and other community representatives in catchment communities proved to be less effective. Whilst the step-down short courses clearly improved communication, the short duration of such training and orientation inevitably meant that full understanding of the complexity and reasons for the training was compromised. The evaluation found that some of the beneficiaries of this shorter training showed a lack of understanding of the term ‘under-supported’ in that they referred only to ‘those who don’t go to clinics’, or to people who fell into categories such as ‘disabled, mentally ill, widows, orphans, or women beaten by husbands’. Whilst it is true that such categories may include women who are under-supported, individuals in these categories may also be well-supported, limiting their usefulness as a means of targeting resources to where they are needed the most. These challenges reinforce the importance of a longer orientation and subsequent reinforcement and follow-up. They also reflect the more general challenges related to the effective provision of in-service training in contexts where health facilities are affected by challenging staffing shortages.

A key feature of the MORE MAMaZ communication training was the reliance not so much on individual training, but rather on the training of teams of staff at the health facilities. This enabled mutual reinforcement and helped to increase staff morale, as health workers began to believe that their supervisors listened to their ideas and concerns. It would be of great benefit if all health workers were oriented in the same way in pre-service training. This is especially important in contexts where health facilities operate in low-resource environments and are affected by high staff turnover.

Despite the clear benefits of the communications training intervention (such as better health worker morale, greater trust in services, a positive impact on domestic social situations, better balance between community services and clinic-based services), the problems facing the development, establishment and maintenance of such an approach are the same as those faced by many health systems. In Zambia, there are inadequate funds available for in-service training and for follow-up supervision and management. Districts are highly dependent on taking advantage of externally funded programs to do what little training they can provide. The majority of in-service training conducted by District Health Teams is piggy-backed on to training funded by external donors. During the implementation timeframe of MORE MAMaZ, despite considerable interest in, and support for, the communications training within the respective DHMTs, none of the districts were able to provide any additional support or expanded training other than that provided within the context of the program itself. During the evaluation process however, possibilities for extending the training were explored with the DHMTs. They also signaled their commitment to using their regular monitoring of clinics and of staff through the staff appraisal process to embed some sort of follow-up of the training. With just a few adjustments to existing staff monitoring mechanisms, they acknowledged that it should be possible to establish some form of on-going assessment of the extent of good communication both among health workers and between health workers and their clients.

The evaluation of the communication training revealed some side-effects that were not fully expected. The increase in the number of women who were willing to talk about domestic social issues in clinic consultations showed that it is possible to contribute through this route to on-going efforts to reduce intimate partner violence. Listening to women and girls affected by violence and giving them confidence to communicate about their problems is a first step, especially in cases where they lack other opportunities to communicate about the difficulties they are facing.

The MORE MAMaZ communications training design was strongly influenced by a study that investigated the relationship between child mortality and lack of support among women within the context of a large health systems strengthening program focused on improving maternal, newborn and child health in northern Nigeria between 2006 and 2014 [9]. Implemented in a context where study populations were generally very poor, had few resources, similar employment opportunities, and the overall culture within which the entire population operated, the study identified a skew of child mortality where 20 percent of survey households had experienced 80 percent of the child deaths. This type of skew has been recognized by demographers for well over one hundred years [15,16,17,18,19,20,21,22,23,24,25]. However, the standard variables used in studies that have explored this skew (e.g., health service availability, birth spacing, availability of services, poverty, education, resources, etc.) have failed to provide sufficient explanation. Responding to this gap in understanding, the Nigeria study tested the hypothesis that social variables relating to the support of women were important in determining the skew of mortality. The hypothesis was that those women who felt least respected and supported by their husbands or families would be the most likely to neglect themselves and their children. This was found to be the case. Health seeking behavior, child mortality, and lack of delivery by trained midwives were all strongly linked to the support the woman received from their husbands or families [9]. The Nigeria study strongly influenced the design of the communications training. Wider implications of this approach are that health programs that aim to reach every woman, adolescent and child need to disaggregate beyond poverty (and other standard variables such as level of education), and place more emphasis on understanding the social factors that influence poor health. Perhaps one of the most important implications is the need to move from a focus on individual women and children, towards a focus on the family unit, as it is the situation of the family and its interactions that have the most significant impact on the health (including the mental health) of each and every family member.

## 5. Conclusions

The impact of the MORE MAMaZ communications training intervention could be seen through improved clinic attendance; improved relevance of clinical diagnosis, treatment, support and referral of under-supported women; improved morale of staff and fewer complaints about staff; and a decrease in intimate partner violence, with a concomitant improvement in the participation and voice of women affected by violence. The fact that a short communications training stimulated Zambian health workers to explore, try to understand and respond to clients’ social situations, indicates that it is possible to operationalize the social determinants of health perspectives in clinical settings. The way in which the communications training was perceived by health workers, DHMTs and community members to have contributed to an increase in poorly-supported women and girls using maternal health services is significant in a context where there is as yet inadequate understanding of how to effectively reach the least-supported individuals. Further testing of the efficacy of this training is warranted in the Zambian context, including in a pre-service training scenario. Should this approach to communications training and inclusion of under-supported women prove to be useful in other contexts, it will provide a valuable contribution to the goals of the Global Strategy for Women’s, Children’s and Adolescent’s Health, with its emphasis on reaching every woman, adolescent and child.

## References

[1] White Ribbon Alliance (2011). Respectful Maternity Care Charter: The Universal Rights of Childbearing Women [Internet].

[2] World Health Organization (2014). The Prevention and Elimination of Disrespect and Abuse during Facility-Based Childbirth [Internet].

[3] Every Woman Every Child (2015). Global Strategy for Women’s, Children’s and Adolescents’ Health (2016–2030).

[4] De Silva M.J., McKenzie K., Harpham T., Huttly S.R. (2005). Social capital and mental illness: A systematic review. J. Epidemiol. Community Health.

[5] Lund C., Breen A., Fisher A.J., Kakuma R., Corrigall J., Joska J.A., Swartz L., Patel V. (2010). Poverty and common mental disorders in low and middle income countries: A systematic review. Soc. Sci. Med..

[6] Patel V., Leon D.A., Walt G. (2001). Poverty, inequality, and mental health in developing countries. Poverty, Inequality and Health: An International Perspective.

[7] Mindes E.J., Ingram K.M., Kliewer W., James C.A. (2003). Longitudinal analyses of the relationship between unsupportive social interactions and psychological adjustment among women with fertility problems. Soc. Sci. Med..

[8] Rahman A., Iqbal Z., Bunn J., Harrington R. (2004). Impact of Maternal Depression on Infant Nutritional Status and Illness. Arch. Gen. Psychiatry.

[9] PRRINN-MNCH (2014). Adjusting Health Strategies to Include Women and Children with the Least Social Support.

[10] Central Statistical Office [Zambia], Ministry of Health [Zambia], ICF International (2015). Zambia Demographic and Health Survey 2013–14.

[11] Zimbabwe Ministry of Health and Child Welfare (Health Education Unit) (1998). Interpersonal Communication: Manual for Trainers of Health Service Providers.

[12] Partnership for Transforming Health Systems Programme (2004). An Interpersonal Communication and Counselling (IPC & C) Skills Training Manual for Health Care Providers.

[13] Bramhall E. (2014). Effective communication skills in nursing practice. Nurs. Stand..

[14] Kureya T., Green C., Soyoola M. (2016). MORE MAMaZ Endline Survey Report.

[15] Meegama S.A. (1980). Socio-Economic Determinants of Infant and Child Mortality in Sri Lanka: An Analysis of Post-War Experience.

[16] Arulampalam W., Bhalotra S. (2003). Sibling Death Clustering in India: Genuine Scarring vs Unobserved Heterogeneity.

[17] Barthélémy K.D., Diallo K. (2002). Geography of child mortality clustering within African families. Health Place.

[18] Das Gupta M. (1990). Death clustering, mother’s education and the determinants of child mortality in rural Punjab, India. What We Know about Health Transition: The Cultural, Social and Behavioural Determinants of Health: The Proceedings of an International Workshop, Canberra, May 1989.

[19] Das Gupta M. (1997). Socio-economic status and clustering of child deaths in rural Punjab. Popul. Stud..

[20] Edvinsson S., Brändström A., Rogers J., Broström G. (2005). High-risk families: The unequal distribution of infant mortality in nineteenth-century Sweden. Popul. Stud..

[21] Guo G. (1993). Use of sibling data to estimate family mortality effects in Guatemala. Demography.

[22] Madise N.J., Diamond I. (1995). Determinants of infant mortality in Malawi: An analysis to control for death clustering within families. J. Biosoc. Sci..

[23] Omariba D.W.R. (2005). Levels, Trends and Correlates of Child Mortality in Kenya: An Exploration into the Phenomenon of Death Clustering.

[24] Ronsmans C. (2005). Patterns of Clustering of Child Mortality in a Rural Area of Senegal. Popul. Stud..

[25] Vandezande M., Moreels S., Koen M. (2010). Explaining Death Clustering: Intergenerational Patterns in Infant Mortality Antwerp 1846–1905.

